# Economic Evaluation of Population-Based *BRCA1/BRCA2* Mutation Testing across Multiple Countries and Health Systems

**DOI:** 10.3390/cancers12071929

**Published:** 2020-07-17

**Authors:** Ranjit Manchanda, Li Sun, Shreeya Patel, Olivia Evans, Janneke Wilschut, Ana Carolina De Freitas Lopes, Faiza Gaba, Adam Brentnall, Stephen Duffy, Bin Cui, Patricia Coelho De Soarez, Zakir Husain, John Hopper, Zia Sadique, Asima Mukhopadhyay, Li Yang, Johannes Berkhof, Rosa Legood

**Affiliations:** 1 Wolfson Institute for Preventive Medicine, CRUK Barts Cancer Centre, Queen Mary University of London, London EC1M 6BQ, UK; li.sun1@lshtm.ac.uk (L.S.); shreeyapatel09@hotmail.co.uk (S.P.); o.evans@qmul.ac.uk (O.E.); f.gaba@qmul.ac.uk (F.G.); 2 Department of Gynaecological Oncology, Barts Health NHS Trust, Royal London Hospital, London E1 1BB, UK; 3 MRC Clinical Trials Unit at UCL, Institute of Clinical Trials & Methodology, Faculty of Population Health Sciences, University College London, London WC1V 6LJ, UK; 4 Department of Health Services Research and Policy, London School of Hygiene & Tropical Medicine, London WC1H 9SH, UK; Zia.Sadique@lshtm.ac.uk (Z.S.); Rosa.Legood@lshtm.ac.uk (R.L.); 5 Department of Epidemiology and Biostatistics, Amsterdam UMC, Vrije Universiteit Amsterdam, 1081 HV Amsterdam, Netherlands; j.wilschut@amsterdamumc.nl (J.W.); h.berkhof@amsterdamumc.nl (J.B.); 6 Departamento de Medicina Preventiva, Faculdade de Medicina FMUSP, Universidade de Sao Paulo, 01246903 Sao Paulo, Brazil; acflopes@usp.br (A.C.D.F.L.); patricia.soarez@usp.br (P.C.D.S.); 7 Centre for Cancer Prevention, Wolfson Institute of Preventive Medicine, Queen Mary University of London, London EC1M 6BQ, UK; a.brentnall@qmul.ac.uk (A.B.); s.w.duffy@qmul.ac.uk (S.D.); 8 School of Public Health, Peking University, Beijing 100191, China; cuibin@bjmu.edu.cn (B.C.); lyang@bjmu.edu.cn (L.Y.); 9 Department of Humanities & Social Sciences, Indian Institute of Technology, Kharagpur, West Bengal 721302, India; dzhusain@gmail.com; 10 Department of Economics, Presidency University, Kolkata 700073, India; 11 Centre for Epidemiology & Biostatistics, Melbourne School of Population & Global Health, Faculty of Medicine, Dentistry & Health Sciences, University of Melbourne, Victoria 3010, Australia; j.hopper@unimelb.edu.au; 12 Tata Medical Centre, Kolkata, West Bengal 700160, India; asima7@yahoo.co.in; 13 Northern Institute for Cancer Research, Newcastle University, Newcastle upon Tyne NE2 4HH, UK

**Keywords:** BRCA, population testing, cost-effectiveness, ovarian cancer, breast cancer, cancer prevention

## Abstract

Clinical criteria/Family history-based *BRCA* testing misses a large proportion of *BRCA* carriers who can benefit from screening/prevention. We estimate the cost-effectiveness of population-based *BRCA* testing in general population women across different countries/health systems. A Markov model comparing the lifetime costs and effects of *BRCA1/BRCA2* testing all general population women ≥30 years compared with clinical criteria/FH-based testing. Separate analyses are undertaken for the UK/USA/Netherlands (high-income countries/HIC), China/Brazil (upper–middle income countries/UMIC) and India (low–middle income countries/LMIC) using both health system/payer and societal perspectives. *BRCA* carriers undergo appropriate screening/prevention interventions to reduce breast cancer (BC) and ovarian cancer (OC) risk. Outcomes include OC, BC, and additional heart disease deaths and incremental cost-effectiveness ratio (ICER)/quality-adjusted life year (QALY). Probabilistic/one-way sensitivity analyses evaluate model uncertainty. For the base case, from a societal perspective, we found that population-based *BRCA* testing is cost-saving in HIC (UK-ICER = $−5639/QALY; USA-ICER = $−4018/QALY; Netherlands-ICER = $−11,433/QALY), and it appears cost-effective in UMIC (China-ICER = $18,066/QALY; Brazil-ICER = $13,579/QALY), but it is not cost-effective in LMIC (India-ICER = $23,031/QALY). From a payer perspective, population-based *BRCA* testing is highly cost-effective in HIC (UK-ICER = $21,191/QALY, USA-ICER = $16,552/QALY, Netherlands-ICER = $25,215/QALY), and it is cost-effective in UMIC (China-ICER = $23,485/QALY, Brazil−ICER = $20,995/QALY), but it is not cost-effective in LMIC (India-ICER = $32,217/QALY). *BRCA* testing costs below $172/test (ICER = $19,685/QALY), which makes it cost-effective (from a societal perspective) for LMIC/India. Population-based *BRCA* testing can prevent an additional 2319 to 2666 BC and 327 to 449 OC cases per million women than the current clinical strategy. Findings suggest that population-based *BRCA* testing for countries evaluated is extremely cost-effective across HIC/UMIC health systems, is cost-saving for HIC health systems from a societal perspective, and can prevent tens of thousands more BC/OC cases.

## 1. Introduction

Around 10–20% of ovarian cancer (OC) [1] and 6% breast cancer (BC) [2] overall are caused by inheritable *BRCA1/BRCA2* mutations. Women carrying *BRCA1/BRCA2* mutations have a 17–44% risk of OC and 69–72% risk of BC until age 80 years [3]. Most of these cancers can be prevented in unaffected *BRCA1/BRCA2* women carriers. Women can opt for risk-reducing salpingo-oophorectomy (RRSO), to reduce OC risk [4]. In *BRCA* women, RRSO reduces OC risk by 79–96% [4,5,6]. Additionally, they can opt for MRI/mammography screening, chemoprevention with selective estrogen-receptor modulators (SERM) or aromatase inhibitors [7]; or risk-reducing mastectomy (RRM) [8,9] to reduce their BC risk [10]. RRM reduces BC risk by 90–95% [8,9]. Mutation identification also enables women to make timely, informed reproductive/lifestyle choices and consider prenatal/pre-implantation genetic diagnosis.

Despite 25 years of *BRCA* testing and effective mechanisms for prevention, current guidelines and access to testing/treatment pathways remain complex and associated with a massive under-utilisation of genetic testing [11]. Only 20% of eligible US women have accessed/undergone genetic testing [11]. A UK analysis shows the huge majority (>97%) of *BRCA* carriers in the population remain unidentified [12]. This highlights substantial missed opportunities for early detection and primary prevention. The current approach uses established clinical-criteria/family-history (FH) based *a priori BRCA* probability thresholds to identify high-risk individuals eligible for *BRCA* testing. These clinical criteria/FH-based criteria are used to calculate mutation probability and have been loosened over the years. Earlier, the threshold for offering *BRCA* testing used to be 20% probability. Most countries/health systems now offer *BRCA* testing at a *BRCA* mutation probability of around 10% [13]. A number of different strategies ranging from standardised criteria to complex mathematical (Empirical/Mendelian) models have been used to calculate mutation probability and are used in clinical practice. However, this requires individuals and health practitioners to recognise and act on a significant FH. *BRCA* carriers, who are unaware of their FH, unappreciative of its risk/significance, not proactive in seeking advice, or lack a strong FH (small families/paternal inheritance/chance) get excluded. Over 50% *BRCA* carriers do not fulfil clinical criteria and are missed [14,15,16,17,18,19,20]. Current detection rates are inadequate to identify all *BRCA* carriers and even doubling detection rates will need 165 years to ascertain the ‘clinically detectable’ proportion of *BRCA* carriers [12]. Why should we wait for decades for people to develop cancer before identifying *BRCA* carriers and unaffected at-risk family members to offer prevention? 

These limitations can be overcome through unrestricted/unselected population based *BRCA* testing. Falling *BRCA* testing costs, advances in computing/bioinformatics, and next-generation sequencing has made this possible. Jewish population studies show this is feasible, acceptable, has high satisfaction (91–95%), significantly reduces anxiety, doesn’t harm psychological well-being or quality of life, and is extremely cost-effective [15,16,21,22]. Pilot general population studies are ongoing in the UK/Canada [23]. However, the potential applicability and scope for primary prevention transcends continents and countries. Health systems, infrastructure, costs, environment, contexts, opportunities, and capacity along with health sector priorities vary considerably across different countries, [24]. Economic evaluations of health interventions, health perspectives and cost-effectiveness thresholds differ amongst countries. Nevertheless, economic evaluation is important to weigh up costs and health effects of alternative health strategies, to help health policy decision making with respect to cost efficiency and resource allocation. For interventions to be sustainable, they need to be cost-effective and affordable. The World Bank separates countries into four income categories using Gross National Income (GNI) per capita (USA dollars): Low-income (LIC: ≤$1025), Lower–Middle Income (LMIC: $1026–$4035), Upper–Middle Income (UMIC: $4036–$12,475), and High Income (HIC: ≥$12,476). In settings of state funded universal health care coverage, the difference between government and societal perspectives is narrower than countries with a limited social security structure/net, where this gap can be significantly larger and consequences considerable. We for the first time evaluate the cost-effectiveness of population-based *BRCA*-testing (compared to clinical-criteria/family-history testing) across multiple countries/health systems: India (LMIC), Brazil (UMIC), China (UMIC), the USA (HIC), the UK (HIC), and the Netherlands (HIC). We present analyses from both health system or payer (here forth called ‘payer’) and societal perspectives.

## 2. Results

The comparison of lifetime costs and quality-adjusted life year (QALYs) of population testing and clinical-criteria/FH testing for women in different countries along with the country-specific incremental cost-effectiveness ratios (ICERs) and willingness-to-pay (WTP) thresholds are given in Table 1. Our results show that from a ‘societal perspective’ (using WHO guidelines), population-based *BRCA* testing is actually ‘cost-saving’ and contributes to better health in HIC of the UK (ICER = $−5,639/QALY; life expectancy gained = 3.0 days), USA (ICER = $−4018/QALY; life expectancy gained = 2.2 days), and The Netherlands (ICER = $−11,433/QALY; life expectancy gained = 2.8 days). It appears potentially cost-effective in UMICs of China (ICER = $18,066/QALY; life expectancy gained = 1.8 days) and cost-effective in Brazil (ICER = $13,579/QALY; life expectancy gained = 3.7 days), but it is not cost-effective in India (ICER = $23,031/QALY; life expectancy gained = 2.5 days) (LMIC) for the base case. 

From a ‘payer perspective’ (using WHO guidelines), population-based *BRCA* testing is ‘highly’ cost-effective compared with clinical criteria/FH-based testing in HIC, with UK-ICER = $21,191/QALY (life expectancy gained = 3.0 days), USA-ICER = $16,552/QALY (life expectancy gained = 2.2 days), and Netherlands-ICER = $25,215/QALY (life expectancy gained = 2.8 days). In UMIC population-based *BRCA* testing is cost-effective with ICER = $23,485/QALY in China (life expectancy gained = 1.8 days) and ICER = $20,995/QALY in Brazil (life expectancy gained = 3.7 days). Population-based *BRCA* testing is not cost-effective in LMIC with ICER = $32,217/QALY in India (life expectancy gained = 2.5 days). 

**Figure 1:**
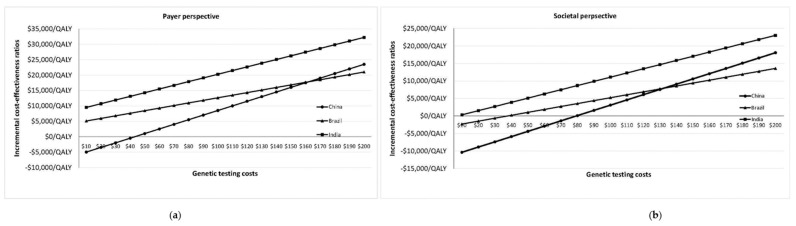
Change in ICER/QALY with varying *BRCA* testing costs in Brazil, China, and India. (**a**) Change in ICER/QALY with varying *BRCA* testing costs in Brazil, China and India from a payer/healthcare perspective. (**b**) Change in ICER/QALY with varying *BRCA* testing costs in Brazil, China, and India from a societal perspective. The graphs depict the change in ICER/QALY at varying costs of BRCA testing for Brazil, China, and India from payer (Figure 1a) and societal (Figure 1b) perspectives. **X axis:** BRCA testing costs in US$; **Y axis:** ICER/QALY.

If we consider local, country-specific guidelines for the UK, USA, and the Netherlands, then population-based *BRCA* testing is cost-effective from the payer perspective (UK-ICER = $24,066/QALY; USA-ICER = $16,552/QALY; Netherlands-ICER = $17655/QALY), and cost-saving from the societal perspective (UK-ICER = −$3543/QALY; USA-ICER = −$4018/QALY; Netherlands ICER = −$3185/QALY). The corresponding values for life expectancy gained are 2.6 days (UK), 2.2 days (USA) and 4.2 days Netherlands. Figure 1a,b plot change in ICER/QALY with varying *BRCA* testing costs in Brazil, China and India for payer and societal perspectives. Population testing becomes potentially cost-effective (from a societal perspective) in India if the *BRCA* testing cost falls to $172/test (ICER = $19,685/QALY) (Figure 1a; Appendix D). *BRCA* testing costs need to reach $95/test (ICER = $19,670/QALY) for cost-effective population testing in India from the payer perspective (Figure 1b; Appendix D). 

The lifetime population impact (reduction in BC and OC cases and deaths; and excess coronary heart disease (CHD)) of offering population *BRCA* testing for the six countries is detailed in Table 2. A population-based *BRCA* testing approach can potentially prevent an additional 2319 to 2666 BC and 327 to 449 OC cases per million women, translating to tens of thousands more BC/OC prevented across the population than the current clinical strategy.

Scenario analyses results are given in Table 3. Different scenarios analysed include no reduction in BC risk from RRSO, nil compliance with hormone replacement therapy (HRT), reduction in RRM and RRSO rates by half, and reduced genetic testing costs of $100. Population-based *BRCA* testing remains cost-effective from payer and societal perspectives in each HIC and UMIC country at their respective WTP thresholds, even without reduction in BC risk from RRSO, no HRT uptake after RRSO, and 50% lower RRM and RRSO uptake rates (Table 3). If the *BRCA* testing costs fell to $100/test, it would be highly cost-effective from the payer perspective and cost-saving (negative ICERs) from the societal perspective for HIC; highly cost-effective from payer/societal perspectives for UMIC, and cost-effective from the societal perspective for India (LMIC). The maximum *BRCA* testing costs for population testing to remain cost-effective from the payer/societal perspectives respectively are in Appendix E. At the 3*GDP WTP threshold, these are: UK = $1254/$1520; USA = $1417/$1577; Netherlands = $1407/$1758; China = $354/$390; Brazil = $493/$582; and India = $95/$172. Using UK/USA/Netherlands guideline-based WTP thresholds, these maximum *BRCA* testing costs are UK = $365, USA = $850–$1010, and Netherlands = $800.

**Figure 2:**
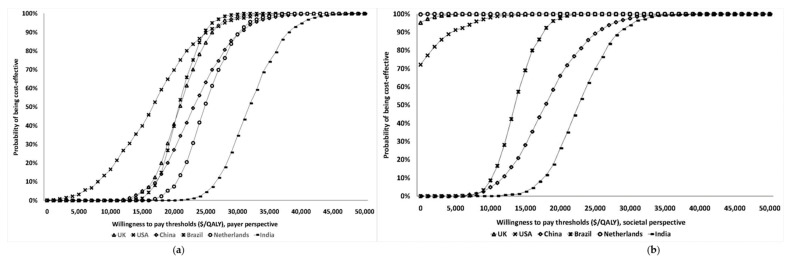
Cost-effectiveness acceptability curves. (**a**) Cost-effectiveness acceptability curve—payer perspective. (**b**) Cost-effectiveness acceptability curve—societal perspective. * The GDP-based (WHO) willingness-to-pay thresholds are $127,969/QALY in the UK, $172,766/QALY in the US, $151,616/QALY in the Netherlands, $46,592/QALY in China, $45,545/QALY in Brazil, and $19,722/QALY in India (Table 2). The country guideline-specific willingness-to-pay thresholds are $42,857/QALY in the UK, $100,000 in the US, and $60,976 in the Netherlands (Table 2). Probabilistic sensitivity analysis in which all model parameters/variables are varied simultaneously across their distributions to further explore model uncertainty. X-axis: Willingness-to-pay thresholds in terms of Cost ($s))/QALY; Y-axis: Proportion of simulations. The results of 1000 simulations were plotted on a cost-effectiveness acceptability curve showing the proportion of simulations (Y-axis) that indicated that the intervention was cost-effective at different willingness-to-pay thresholds (X-axis). Separate curves are plotted for the UK, USA, Netherlands, China, Brazil, and India, with different analyses provided for both payer (Figure 2a) and societal (Figure 2b) perspectives.

Results of the one-way sensitivity analysis indicate that model outcomes are not impacted much by treatment costs, utility scores, mutation prevalence, and probabilities (Appendix E). The variable with the maximum effect on ICERs is the cost of *BRCA* testing. Probabilistic sensitivity analysis (PSA) results (Figure 2) show that at the WTP thresholds in each country, a population-testing strategy is cost-effective compared to clinical-criteria/FH-testing strategy from both the payer and societal perspectives for HIC and UMIC but not LMIC countries evaluated. The PSAs were highly cost-effective for the evaluated HIC and UMIC countries. All (100%) simulations are cost-effective at the guideline-specific thresholds for the UK/USA/Netherlands from payer and societal perspectives. For the 3*GDP-based WTP threshold for China/Brazil/India, 100%/100%/22.2% for the societal perspective and 100%/100%/0% simulations for the payer perspective were cost-effective (Figure 2a,b). However, a population strategy becomes cost-effective in India (LMIC) at $172/test. At the country-specific WTP thresholds for UK/USA/Netherlands, 84.9%/100%/98.5% of simulations for the payer perspective were cost-effective, and 100% simulations for the societal perspective were cost-effective for all three countries).

## 3. Discussion

For the first time, we explore the cost-effectiveness of population-based *BRCA* testing across countries from HIC, UMIC and LMIC health systems. We show that population-based *BRCA* testing is extremely cost-effective across HIC/UMIC health systems assessed and is potentially cost-saving for HIC health systems (UK/USA/Netherlands) if analysed from a societal perspective. Societal perspective analyses are associated with lower ICER/QALY than the payer perspective, as it incorporates additional costs linked to productivity loss. There is increasing recognition of the importance and need for economic cost-effectiveness evaluations to conform to the societal perspective and is recommended by WHO/international bodies. This is particularly important in middle/lower–income countries that lack a robust/comprehensive state-funded social security system. However, some countries such as the UK only consider a payer perspective when making health policy. 

A population-based *BRCA* testing approach can potentially prevent an additional 57,708/269,089/15,181/1,050,314/156,299/692,571 BC cases and 9727/43,817/2557/154,756/25,170/97,659 OC cases in the UK/USA/Netherlands/China/Brazil/India respectively (Table 2) compared to the current clinical strategy. Given the huge under-utilisation of *BRCA* testing along with limited access and uptake associated with current treatment pathways [11,12], one could postulate that the benefit could be even higher. Our findings are important, as we show that a new population-based approach can have much broader global applicability and a far greater impact on BC/OC burden in the population than current treatment strategies. Cost-effectiveness analyses are necessary to guide policy decisions on healthcare resource allocation. Our findings support a change in paradigm toward population testing to maximise OC/BC prevention and highlights a need for further implementation research in this area. 

Our results are sensitive to the cost of testing, particularly in LMIC countries. *BRCA* testing costs need to fall further for population testing to be cost-effective in LMIC countries. In India, it would become potentially cost-effective at $172/test. Although our base case analysis uses costs higher than this, we are aware of Indian providers who offer *BRCA* testing for around $140/test. Genetic testing costs have fallen considerably over the last 5 years and remain on a downward trajectory. While we have used a standard cost for *BRCA* testing that is currently available across countries, some providers may charge more than this. Our analysis of maximum cost(s) of *BRCA* testing for a population testing strategy remaining cost-effective (Appendix D) shows that these lie above what is charged by a number of providers today.

The precise definition of an appropriate cost-effectiveness threshold remains an important issue of ongoing debate. While this has been clearly defined in some (particularly HIC) health systems, a WHO-CHOICE 3*GDP threshold is considered too high by some, as it ignores opportunity costs [30]. Additionally, whilst cost-effectiveness is a key factor for allocating health budgets, it needs to be considered along with context-specific local issues, affordability, budget impact, fairness, and feasibility [31]. Some advocate against a single fixed threshold and recommend a range of thresholds for different contexts. The Norwegian health system prioritises interventions based on health benefit, resource implications, and health loss to the beneficiary if the intervention was absent (higher priority for higher health loss to the beneficiary) [32]. We provide a range of cost estimates for *BRCA* testing linked to varying potential cost-effectiveness thresholds (ICER/QALY) from payer and societal perspectives to help decision makers in UMIC and LIC. This is important, as the main model parameter impacting the overall result is the cost of *BRCA* testing (Figure 1a,b).

Our analysis has several advantages. We follow the transparency principle to facilitate the interpretation of methodology and results and use current standard of care or best practice as the comparator for measuring costs and effects. As per NICE recommendations, we use QALYs to measure health outcomes, which captures both length of life and quality of life and is generalisable across disease states. Our economic evaluation uses a lifetime horizon that is long enough to capture all costs and effects relevant to the decision problem. Additionally, costs and effects are discounted to reflect their value at the time of decision making, ensuring that the potential time preferences of the relevant population are accounted for. Our base case reflects direct health-care costs and health outcomes, and our analysis includes a societal perspective. We explore heterogeneity through scenario analyses and uncertainty and variability through extensive one-way/PSA analyses, as recommended. Our results remain robust at parameter extremes on one-way analysis (Appendix E) and with PSA (Figure 2). Our analysis uses PPP (purchasing power parity), which is a mechanism for accounting for different relative costs of goods when undertaking a comparative analysis of expenditures and incomes in different countries. Besides OC/BC outcomes, we also included excess CHD deaths from premenopausal oophorectomy [33] and incorporate costs for HRT, excess heart disease, bone health monitoring, and treatment. Our costs also include pre-test counselling for all and post-test genetic counselling for pathogenic mutations and VUS. 

Similar to other modelling studies, our study has some limitations. In line with earlier analyses in high-risk and low-risk women, our base case analysis assumes a reduction in BC risk with premenopausal oophorectomy. However, recently, there has been uncertainty around the benefit of BC risk reduction from RRSO. Nonetheless, our scenario analysis shows cost-effectiveness in HIC/UMIC even without BC risk reduction (Table 3). We use established surgical prevention rates from HIC in the base-case analysis (Table 4). However, RRM/RRSO rates vary, and lower rates are reported in some populations [34]. The uptake of breast screening, chemoprevention, and risk-reducing surgery may also be influenced by socioeconomic, demographic, and cultural factors and may vary across populations [34]. Rates of screening and preventive interventions have also increased with time. Higher rates are reported in the last 10 years compared to earlier decades, as knowledge and awareness of these issues has improved. Rates could be lower in carriers ascertained from population testing, particularly in the absence of cancer burden in the family. More prospective data on the uptake of surgical prevention following population-based testing will be needed. Our scenario analyses confirm cost-effectiveness for both payer and societal perspectives, even at half of standard surgical prevention rates (Table 3). Although we incorporate a disutility for RRSO and RRM in the analysis, these procedures have potential complication rates of around 3–4% and 21%, respectively [35,36]. This needs to be part of the informed consent and decision-making process. While RRSO has been reported to have high satisfaction rates, less cancer worry, and no detriment in generic quality of life; poorer sexual function despite HRT use has been found [37]. RRM has an adverse association with body image and sexual pleasure but not with sexual activity/habit/discomfort, anxiety/depression, or generic quality of life, and overall satisfaction rates are good. Countries such as India and China lack established national breast cancer screening programmes. The uptake of mammograms is much lower in these countries. The cost-effectiveness of population testing may be higher for these countries than estimated, as the implementation of these interventions in *BRCA* carriers are likely to be more beneficial in the absence of routine mammograms in the population. In our analysis, while we included productivity loss, we did not include all indirect costs in the analysis. This may be a limitation. However, including additional indirect costs would improve cost-effectiveness, so our analysis is conservative in that respect. While our analysis covers some important/key countries across different income groups, it does not cover most countries, and therefore, these results are not generalisable globally to all countries across different (HIC/UMIC/LMIC) income groups. While the countries represented in this analysis are from four continents—North America, South America, Europe, and Asia—we do not have representation from Africa or Australia. The populations of countries in our analysis contribute approximately 45% to the global population. 

Population-based *BRCA* testing implementation studies have been completed in the Jewish population [15,21,22,38], and pilot ones are being undertaken in the UK and Canadian general populations [23]. For population testing to be feasible, newer approaches for delivering pre-test information will be needed to facilitate informed decision-making. These will need to be country/region or context-specific. The best modality to deliver pre-test education within the population testing setting remains unresolved. We do not feel there will eventually be a one-size-fits-all model. Although we have costed for pre-test counselling for all in our analyses, whether formal pre-test counselling will be needed for all in the future remains uncertain. Israeli and Canadian Jewish population studies provided only ‘pre-test information’ and post-test genetic counselling for *BRCA* carriers, with >90% satisfaction rates [39,40]. An Australian Jewish population [41] and a UK general population study have demonstrated the feasibility of an online web-based decision aid (along with an optional telephone helpline) pre-test education and consent process [42].

A strategy for the management of variants of unknown significance (VUS) is important and will need developing. People have raised concerns at unnecessary treatment or screening/preventive intervention(s) being undertaken for VUS alone. However, VUS are currently identified through routine clinical testing, too. There is clear acceptance in clinical practice that for a VUS (class-3 variant), no clinical action should be taken based on that variant alone [43]. A key presumption inherent in a public health screening strategy is that it is not designed to identify ‘all’ individuals with disease, but the large/significant proportion of individuals in a clinically efficient and cost-effective manner. Therefore, some suggest an alternative option of not providing VUS results within a population-testing context [14]. We incorporate a cost for VUS counselling and management in our analysis.

Chronic disease accounts for 90% US Medicare and 70% UK health care expenditure and is a major challenge facing most health systems, with cancer being its second commonest cause. Between 2006 and 2016, the average annual age-standardised incidence rates for all cancers increased in 130 of 195 countries [44]. The leading cause for women is BC: 1.7 million cases, 535,000 deaths, 14.9 million disability adjusted life-years (DALYs) [44]. Globally breast/ovarian cancers in women are predicted to increase by 46.5%/47% and cancer deaths are predicted to increase by 58.3%/58.6% respectively over the next 20 years [45]. Population testing for *BRCA* genes can significantly increase *BRCA* carrier detection rates for maximising prevention and reducing cancer burden. It can also serve as an initial model, which subsequently informs the potential applicability of a population testing risk-stratification strategy for other cancer genes and other chronic diseases. 

While developing an approach towards implementing population-based *BRCA*-testing, it is important to bear in mind the principles of population testing of disease. These were initially proposed by Wilson and Jungner [46]. Updated criteria have been suggested by the UK National Screening Committee [47], Khoury [48], the CDC (ACCE model) [49], and Burke and Zimmerman (Public Health Foundation) [50]. Analytic validity, clinical validity, clinical utility, and associated ethical, legal, and social implications remain key principles of the ACCE model, providing a framework for evaluating the applicability of a genetic test [49]. In our study, we focussed on *BRCA* testing, as testing for these genes has well-established clinical utility fulfilling the ACCE principles. Multigene panel testing is widely available in current clinical practice. We are against indiscriminate large-scale commercial panel testing without well-established clinical benefit/utility in the population-testing context. The low incidence of moderate penetrance genes, poor precision, and wide confidence intervals around prevalence and penetrance estimates require more data on the clinical significance of pathogenic variants in multigene panels, and these are reasons against currently implementing large multigene panel testing in the general population [51,52]. The USPSTF currently recommends against population testing in the general population [51]. More data are needed on the ‘E’ (Ethical, legal, and social implications) of a population-based *BRCA* testing approach across different populations and health systems. There is an urgent need for multiple implementation studies across countries for evaluating general population *BRCA* testing and to develop local/regional and context-specific implementation pathways. These studies will need to provide prospective data on the impact of population testing on psychological well-being, quality of life, long-term health behaviour, socio-ethics, and lifestyle outcomes. A number of challenges and logistic hurdles will need to be overcome, including varying levels of workforce expansion/upskilling and the reorganisation of health services infrastructure. These include increasing public and health-professional awareness, establishing/expanding laboratory testing infrastructure, expanding downstream management pathways, and involving general practitioners, genetics services, gynaecologists, and breast clinicians/services. A framework/structure for data management and legal and regulatory protections will need to be established. These changes will need to be system/country and context-specific. 

## 4. Materials and Methods 

**Figure 3:**
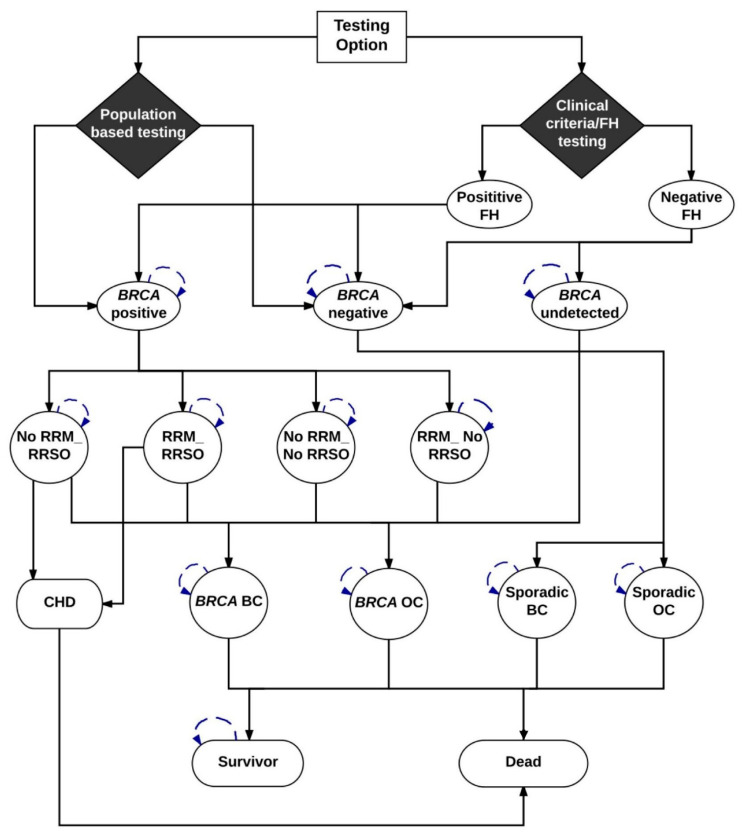
BC, breast cancer; CHD, coronary heart disease; FH, family history; OC, ovarian cancer; RRM, risk-reducing mastectomy; RRSO, risk-reducing salpingo-oophorectomy.

We developed a Markov model (Figure 3) (TreeAge-Pro-2018 Williamson, MA, USA) to compare the lifetime costs and effects of *BRCA1/BRCA2* testing all general population women ≥30 years compared with clinical-criteria/FH-based testing. We describe separate analyses for populations in the UK, USA, Netherlands, China, Brazil, and India using both payer and societal perspectives. While some countries only consider a payer perspective, a societal perspective is recommended by the WHO and other international bodies [53]. In the model, all women ≥30 years in the Population testing arm and only those fulfilling clinical/FH criteria in the Clinical-Criteria/FH-based testing arm undergo genetic testing for *BRCA* mutations. We include pre-test counselling for all and assume a 70% uptake of genetic testing (from the published literature) [22]. We include the cost of post-test counselling for mutation carriers as well as the cost of post-test counselling for those with variants of uncertain significance (VUS). We assume a VUS prevalence of 2% [54]. Model probabilities are described in Table 4, Appendix A, and costs are outlined in Appendix B. *BRCA* carriers identified are offered RRSO to reduce OC risk [4] and MRI/mammography screening, chemoprevention with SERM or RRM [8] to reduce their BC risk [10]. OC screening is excluded given the lack of mortality benefit. Women undergoing RRSO receive hormone replacement therapy (HRT) until 51 years. We include the costs of bone health monitoring and dual energy X-ray scans. We incorporate the excess risk and mortality from coronary heart disease (CHD) after premenopausal RRSO for women who do not take HRT (absolute mortality increase = 3.03%) [33]. Associated costs are modelled over an individual’s lifetime. The Markov cycles’ run depends on life expectancy and these are different across countries (starting from age 30): UK = 53 cycles, US = 52 cycles, Netherlands = 53 cycles, China = 48 cycles, Brazil = 49 cycles, and India = 38 cycles. Cancer incidence is estimated by summing the probabilities of pathways ending in OC or BC. 

### 4.1. Probabilities

The model probabilities for different pathways are given in Table 4, and a detailed explanation is given in Appendix A. The age-specific incidence of BC and OC among general population women is obtained from Cancer Research UK [55,56], USA Cancer Statistics [57], and the International Agency for Research on Cancer (GLOBOCAN-2018) [58]. The BC/OC incidence for *BRCA1/BRCA2* carriers is obtained from the literature [3]. 

Figure 3 is a schematic diagram showing the Markov model structure for population and clinical-criteria/family-history (FH)-based *BRCA1/BRCA2* testing. In the Population testing arm, all women ≥30 years old are offered *BRCA1/BRCA2* testing and get classified as *BRCA*-positive and *BRCA*-negative. *BRCA* mutation carriers identified are offered options of risk-reducing mastectomy (RRM) and risk-reducing salpingo-oophorectomy (RRSO). Depending on the probability of *BRCA* women undertaking RRM and/or RRSO (+/− chemoprevention), they are placed into different health states and then progress to either *BRCA*-associated breast cancer (BC) or *BRCA*-associated ovarian cancer (OC). All women undergoing RRSO have an increased risk of fatal coronary heart disease (CHD). In addition, they have a probability of dying from the background all-cause mortality. Hence, patients in the model can go from intervention to death without ever developing breast cancer, ovarian cancer, or coronary artery disease. Patients can move from healthy state to death as they have a probability of dying from the background all-cause mortality. *BRCA*-positive women who do not progress or die would stay in the health states and undertake the next cycle. *BRCA1/BRCA2*-negative women progress to sporadic non-*BRCA* OC or non-*BRCA* BC based on the age-dependent probabilities. They also have a probability of dying from the background all-cause mortality. Women do not progress or die would stay in the health states to undertake the next cycle. 

In the Clinical criteria/FH arm, only women whose FH fulfil current clinical criteria (based on current guidelines) undergo *BRCA1/BRCA2* genetic testing and get classified as *BRCA*-positive and *BRCA*-negative. Women with a negative FH are either *BRCA* negative or have an undetected *BRCA* mutation. Options of RRM and RRSO and disease progression for identified *BRCA* mutation carriers and disease progression for *BRCA* negative women are the same as those in the population testing arm and are described above. All women undergoing RRSO have an increased risk of fatal coronary heart disease (CHD). Undetected *BRCA* women are not offered RRM or RRSO. Depending on the baseline risk (no risk-reducing options), they progress to *BRCA*-associated BC or *BRCA*-associated OC. In addition, they have a probability of dying from the background all-cause mortality. Hence, patients in the model can go from intervention to death without developing breast cancer, ovarian cancer, or coronary artery disease. Patients can move from healthy state to death as they may die from the background all-cause mortality. Women who do not progress or die stay in the health state of *BRCA* undetected and undertake the next cycle.

Progression through the model is dependent on the probabilities provided in Table 4.

### 4.2. Costs

The analysis was conducted from both a payer perspective and societal perspective. All costs are reported at 2016 USA dollars, which was converted by purchasing power parity (PPP) factor [28]. PPP reflects the value of a country’s currency required to purchase equivalent amounts of goods and services in the domestic market as the USA dollar would buy in the USA. Thus, it is used to translate and compare costs of goods/services between countries using the USA dollar as a common reference point. For comparison, we convert values in all other country currencies (£s, €s, ¥, ₹, R$) to $ (USA) using the purchasing power parity (PPP) factor [28]. In line with the National Institute of Health and Care Excellence (NICE) recommendations, future healthcare costs not associated with BC/OC/heart disease were not considered [25]. We collected primary data on relevant direct medical costs from the Urban Basic Medical Insurance Database in China [65]; the Dutch Healthcare Authority (NZA) in Netherlands; Management System of Procedures/Medical drugs/Orthotics/Prosthetics/Special Materials (SIGTAP) [66], the Health Price Bank (BPS) [67], and Chamber of Regulation of the Market of Medicines (CMED) [68] in Brazil; and an accredited cancer centre (Tata Medical Centre) in India (details in Appendix B). Costing data were obtained from published national health service (NHS) reference costs for the UK [69,70] and published literature for the USA (details in Appendix B). We adopted a standard internationally available *BRCA* testing cost (US $200) for our base case and explored the impact of change in testing costs on the overall results in the sensitivity analyses.

The retirement ages for females are 65 in the UK, 62 in the USA, 50–55 in China, 60 in Brazil, 68 in Netherlands, and 60–65 in India. We used the lower values of the retirement age ranges in China and India to get the conservative estimates of productivity loss. The female labour force participation rates are 56.77% in the UK, 55.99% in the USA, 62.03% in China, 53.32% in Brazil, 58.02% in the Netherlands, and 27.45% in India, which were obtained from the World Bank [71]. For the hourly wage rates across countries, see Appendix C. Additionally, we categorised costs due to productivity loss (for details: see Appendix C) as three subcomponents: (1) temporary disability due to short-term work absences following diagnosis, (2) permanent disability from reduced working hours following return to work or workforce departure; and (3) premature mortality due to death before retirement [72]. We estimated temporary disability as time absent from work multiplied by age-specific gross earnings. We calculated productivity costs due to permanent disability by applying age-specific gross earnings to the reduction in working hours, or the number of working hours in cases of permanent workforce departure, until retirement age. Regarding productivity loss from premature mortality, we assumed that without cancer, the productive capacity of an individual would continue from the age of diagnosis until the age of retirement. We multiplied the projected years of life lost by the age-specific gross earnings for the remainder of the working life to generate monetary estimates (see Appendix C). While we included productivity loss, we did not include all indirect costs in the analysis.

### 4.3. Life Years

Lifetime tables from each country were used to model the lifetime health outcomes, and these were obtained from the World Health Organisation (WHO) [73]. The median ages for RRM and RRSO in unaffected *BRCA* carriers were assumed to be 37 and 40 years [60]. BC and OC survival were modelled using five-year survival data from the CONCORD global surveillance of cancer survival [74]. No significant overall long-term survival differences between germ-line and sporadic BC/OC have been found [75,76,77]. After five years, the probability of death was assumed to be the same as that of the general population. Modelling estimated the number of BC cases, OC cases, BC deaths, OC deaths, and excess CHD deaths per million women aged 30 years in the six countries, and it calculated the number of cases prevented and deaths prevented. The actual numbers of cases prevented and deaths prevented were estimated based on the number of female population aged over 30 years in the six countries [29]. 

### 4.4. Quality-Adjusted Life Years (QALY)

QALYs are recommended by NICE as the appropriate summary measure of health effects for economic evaluation. Utility scores multiplied by life years provides QALYs. QALY = (survival in life years) x (utility score). Utility score is an adjustment for quality of life. It is an indication of individual preferences for specific health states where 1 = perfect health and 0 = death. The utility scores for early, advanced, recurrent, and end-stage breast cancer are 0.71, 0.65, 0.45, and 0.16 [78]. The utility scores used for early, advanced, recurrent, and end-stage OC are 0.81, 0.55, 0.61, and 0.16, respectively [79]. Additionally, utility scores used for RRM is 0.88 (SD = 0.22) and RRSO is 0.95 (SD = 0.10) [80].

### 4.5. Analysis

The Markov model is illustrated in Figure 3. Model outcomes include OC, BC, and excess deaths from CHD. Future costs and health effects are discounted at WHO-recommended 3% rate for the WHO analyses [81] and at country-recommended rates for country-specific analyses (see Table 1). The lifetime costs and QALYs were estimated in both population-testing and clinical-criteria/FH-testing arms. The incremental cost-effectiveness ratio (ICER) was calculated by dividing the difference in cost by the difference in health effects between these two strategies. ICER = (Cost^Population-Testing^–Cost^Criteria/FH-testing^)/(Effect^Population-Testing^–Effect^Criteria/FH-testing^). The potential population impact was estimated by calculating the additional reduction in BC and OC incidence/deaths obtained through *BRCA* testing women aged >30 years. We present analyses using a range of cost-effectiveness thresholds. For all countries, we present the initial WHO recommendation of three times gross domestic product (GDP) per capita (threshold of being cost-effective) and one-time GDP per capita (threshold for being highly cost-effective) [82]. For countries (UK [25], USA [26], Netherlands [27]) with specific health economic willingness-to-pay (WTP) threshold guidelines, we also present analysis using those guidelines: UK = £20,000–30,000 [25]; USA = $50,000–100,000 [26]; Netherlands = €20,000–50,000. [27] Additionally, given the lack of a clear established threshold, we evaluate changes in ICER/QALY with *BRCA* testing costs for China, Brazil, and India to identify the *BRCA* testing cost threshold for a given economic cost-effectiveness threshold. We use $ (USA) conversion with PPP for comparison [28]. 

We also explored a number of scenario analyses, including: (1) no BC risk reduction from RRSO (p9 = 1); (2) no HRT uptake (p13 = 0); (3) 50% reduction in RRM uptake; (4) 50% reduction in RRSO uptake; (5) lower *BRCA*-testing costs of $100; and (6) the maximum genetic testing costs at which population *BRCA* testing remains cost-effective (see Table 3, Appendix D). In the one-way sensitivity analysis, each parameter is varied to evaluate their individual impact on results. Probabilities and utility scores were varied according to 95% confidence intervals or ranges where available or by +/−10%. Costs were varied by +/−30%. Probabilistic sensitivity analysis (PSA) was undertaken, and parameters varied simultaneously across their distributions. Costs were specified as having a Gamma distribution, quality of life was specified as having a log-normal distribution, and probability was specified as having a beta distribution, as recommended [83]. A cost-effectiveness acceptability curve was used to plot the results of 1000 simulations for each country, showing the probability of population-based *BRCA* testing being cost-effective at different WTP thresholds. Different curves were generated for payer and societal perspectives.

## 5. Conclusions

The increasing societal awareness and acceptability of genetic testing, falling costs, computational advancements, and technological advancements provides the ability to implement large-scale population testing. We have demonstrated the potential cost-effectiveness of *BRCA* testing on a much broader scale in the general population and across a number of health systems. This is cost-effective for HIC and UMIC health systems and can prevent tens of thousands more BC and OC than the current clinical strategy. Such an approach can bring about a new paradigm for improving global cancer prevention. Context-specific implementation strategies and pathways for population testing need to be developed. A number of implementation studies providing data on the impact of population *BRCA* testing on real-world outcomes are needed. All this is essential for population genomics to achieve its potential for maximising early detection and cancer prevention. 

## Figures and Tables

**Table 1:** Baseline analysis.

**Baseline Analysis Based on WHO Guidelines, Using GDP-Based Thresholds**														
	**Population-Based Testing**				**^#^ FH-Based Testing**				**ICER**				**WTP Threshold ($/QALY)**	
	**Health Effects**		**Costs**		**Health Effects**		**Costs**		**Cost/LY**		**Cost/QALY (95% Credible Intervals)**		1*GDP per Capita	3*GDP per Capita
	**LY**	**QALY**	**Payer**	**Societal**	**LY**	**QALY**	**Payer**	**Societal**	**Payer**	**Societal**	**Payer**	**Societal**		
**UK**	25.67	25.62	2543	18,568	25.66	25.61	2336	18,623	25,530	−6794	21,191 (14,857, 29,619)	−5639 (−11,880, 1895)	42,656	127,969
**USA**	25.23	25.18	7250	21,951	25.22	25.17	7122	21,982	20,997	−5097	16,552 (4435, 30,280)	−4018 (−15,947, 8764)	57,589	172,766
**Netherlands**	25.86	25.81	2478	24,642	25.85	25.80	2239	24,750	30,587	−13,868	25,215 (18,193, 34,069)	−11,433 (−18,054, −3689)	50,539	151,616
**China**	20.70	20.69	820	7687	20.70	20.68	665	7568	30,788	23,684	23,485 (13,947, 36,162)	18066 (8683, 30,653)	15,531	46,592
**Brazil**	24.54	24.51	834	6314	24.53	24.49	586	6153	24,496	15,844	20,995 (15,707, 27,953)	13,579 (8561, 20,180)	15,182	45,545
**India**	18.17	18.16	634	30,968	18.17	18.15	369	30,779	39,473	28,218	32,217 (23,982, 42,786)	23,031 (15,107, 22,112)	6574	19,722
**Country-Specific Analysis Based on Local Health Economic Guidelines Where they Exist**														
	**Population-Based Testing**				**^#^ FH-Based Testing**				**ICER**				**WTP Threshold ($/QALY)**	
	**Health effects**		**Costs**		**Health effects**		**Costs**		**Cost/LY**		**Cost/QALY (95% Credible Intervals)**			
	**LY**	**QALY**	**Payer**	**Societal**	**LY**	**QALY**	**Payer**	**Societal**	**Payer**	**Societal**	**Payer**	**Societal**		
**UK ^∏^**	23.55	23.51	2263	16,570	23.55	23.50	2053	16,601	29,273	−4309	24,066 (16,407, 33,590)	−3543 (−10452, 4901)	28,471	42,857
**USA**	25.23	25.18	7250	21,951	25.22	25.17	7122	21,982	20,997	−5097	16,552 (4435, 30,280)	−4018 (−15947, 8764)	50,000	100,000
**Netherlands ^∫^**	34.58	34.51	1968	19,109	34.57	34.49	1725	19,153	20,796	−3752	17,655 (12,948, 23,766)	−3185 (−7568, 2319)	24,390	60,976

ICER: incremental cost-effectiveness ratio, LY—life years, QALY—quality-adjusted life years, FH—family history, GDP—gross domestic product. **^#^** Reference Strategy, Costs are given in $ WTP: willingness to pay. This reflects the different cost-effective thresholds for different countries. For GDP-based thresholds: Three times GDP per capita is the threshold for being cost-effective and one time GDP per capita is the threshold for being highly cost-effective. Discount rate is 3% for costs and health effects (LYs and QALYs). For country-specific thresholds: For the UK, this is £20,000 to £30,000 [25]; For the USA, this is $50,000 to $100,000 [26]; For the Netherlands, this is: €20,000 to €50,000 [27]. Values in £s and €s have been converted to $ using PPP (purchasing power parity) [28]. ^∏^ For the UK, the discount rate is 3.5% for costs and health effects as per National Institute of Health and Care Excellence (NICE) economic evaluation guidelines [25]. ^∫^ For the Netherlands, the discount rate is 4% for costs and 1.5% for QALYs as per Dutch health economic analysis guidelines. Perspective: Dutch guidelines recommend a societal perspective. UK NICE guidelines recommend a payer perspective [25]. (See Appendix D for details and references). 1*GDP means 1 × GDP; 3*GDP means 3 × GDP

**Table 2:** Lifetime population impact of offering genetic testing for the population.

	Population-Based Testing		FH-Based Testing		Difference	
	Per Million	Actual	Per Million	Actual	Per Million	Actual
**UK (female population over 30 years = 21,760,299)**						
BC cases	112,014	2,437,458	114,666	2,495,166	−2652	−57,708
OC cases	15,822	344,291	16,269	354,018	−447	−9727
BC deaths	12,985	282,557	13,258	288,498	−273	−5941
OC deaths	278	6049	550	11,968	−272	−5919
Excess CHD deaths	17	370	0	0	17	370
**USA (female population over 30 years = 101,428,241)**						
BC cases	106,431	10,795,109	109,084	11,064,198	−2653	−269,089
OC cases	9985	1,012,761	10,417	1,056,578	−432	−43,817
BC deaths	8113	822,887	8285	840,333	−172	−17,446
OC deaths	235	23,836	475	48,178	−240	−24,343
Excess CHD deaths	17	1724	0	0	17	1724
**Netherlands (female population over 30 years = 5,694,479)**						
BC cases	111,732	636,256	114,398	651,437	−2666	−15,181
OC cases	10,964	62,434	11,413	64,991	−449	−2557
BC deaths	11,822	67,320	12,072	68,744	−250	−1424
OC deaths	277	1577	542	3086	−265	−1509
Excess CHD deaths	17	97	0	0	17	97
**China (female population over 30 years = 422,831,894)**						
BC cases	27,062	11,442,677	29,546	12,492,991	−2484	−1,050,314
OC cases	3862	1,632,977	4228	1,787,733	−366	−154,756
BC deaths	3728	1576317	4015	1,697,670	−287	−121,353
OC deaths	163	68922	369	156,025	−206	−87,103
Excess CHD deaths	12	5074	0	0	12	5074
**Brazil (female population over 30 years = 58,670,634)**						
BC cases	66,227	3,885,580	68,891	4,041,879	−2664	−156,299
OC cases	5358	314,357	5787	339,527	−429	−25,170
BC deaths	12,901	756,910	13,421	787,419	−520	−30,509
OC deaths	271	15,900	539	31,623	−268	−15,724
Excess CHD deaths	17	997	0	0	17	997
**India (female population over 30 years = 298,650,697)**						
BC cases	13,713	4,095,397	16,032	4,787,968	−2319	−692,571
OC cases	2826	843,987	3153	941,646	−327	−97,659
BC deaths	3796	1,133,678	4391	1,311,375	−595	−177,697
OC deaths	168	50,173	429	128,121	−261	−77,948
Excess CHD deaths	8	2389	0	0	8	2389

BC—breast cancer, CHD—coronary heart disease, FH—family history, OC—ovarian cancer. The female population data is obtained from the World Bank [29]. We used the modelling to estimate the number of BC cases, OC cases, BC deaths, OC deaths, and excess CHD deaths per million women aged 30 years in the six countries and calculated the number of cases prevented and deaths prevented. The actual numbers of cases prevented and deaths prevented were estimated based on the number of female population aged over 30 years in the six countries [29].

**Table 3:** Scenario analysis.

	Population-Based Testing				FH-Based Testing				ICER				WTP	
	Health Effects		Costs		Health Effects		Costs		Cost/LY		Cost/QALY		GDP per Capita	3*GDP per Capita
	LY	QALY	Payer	Societal	LY	QALY	Payer	Societal	Payer	Societal	Payer	Societal		
**Scenario: No reduction in breast cancer risk from RRSO (P9 = 1)**														
**UK ^†^**	25.67	25.62	2550	18,589	25.66	25.61	2336	18,626	27,692	−4729	23,188	−3960	42,656	127,969
**USA ^‡^**	25.22	25.18	7273	21,982	25.22	25.17	7125	21,986	25,474	−565	20,318	−450	57,589	172,766
**Netherlands ^∫^**	25.86	25.81	2483	24,668	25.85	25.80	2240	24,754	32,834	−11,559	27,318	−9617	50,539	151,616
**China**	20.70	20.69	825	7693	20.70	20.68	666	7569	32,874	25,745	25,401	19,892	15,531	46,592
**Brazil**	24.54	24.51	837	6321	24.53	24.49	586	6154	26,175	17,447	22,577	15,049	15,182	45,545
**India**	18.17	18.16	637	30,974	18.17	18.15	370	30,779	41,333	30,125	34,019	24,795	6574	19,722
**Scenario: No compliance with HRT (P13 = 0)**														
**UK ^†^**	25.67	25.62	2542	18,569	25.66	25.61	2335	18,623	26,315	−6954	21,707	−5736	42,656	127,969
**USA ^‡^**	25.22	25.18	7250	21,951	25.22	25.17	7122	21,982	21,997	−5280	17,173	−4122	57,589	172,766
**Netherlands ^∫^**	25.86	25.81	2477	24,647	25.85	25.80	2239	24,751	31,629	−13,869	25,897	−11,356	50,539	151,616
**China**	20.70	20.69	812	7678	20.70	20.68	664	7566	29,975	22,722	22,750	17,246	15,531	46,592
**Brazil**	24.54	24.51	833	6312	24.53	24.49	586	6153	24,932	16,077	21,296	13,732	15,182	45,545
**India**	18.17	18.16	623	30,957	18.17	18.15	367	30,777	38,327	26,995	31,242	22,005	6574	19,722
Scenario: Half RRM uptake (p2 = 0.235) *														
**UK ^†^**	25.67	25.62	2545	18,590	25.66	25.61	2336	18,627	27,301	−4834	22,648	−4010	42,656	127,969
**USA ^‡^**	25.22	25.18	7265	21,978	25.22	25.17	7125	21,987	24,248	−1503	19,122	−1185	57,589	172,766
**Netherlands ^∫^**	25.86	25.81	2480	24,671	25.85	25.80	2240	24,755	32,616	−11,449	26,879	−9435	50,539	151,616
**China**	20.70	20.69	826	7695	20.70	20.68	666	7569	33,440	26,362	25,453	20,066	15,531	46,592
**Brazil**	24.54	24.51	838	6324	24.53	24.49	587	6155	26,622	17,938	22,762	15,337	15,182	45,545
**India**	18.17	18.16	620	30,959	18.17	18.15	367	30,777	39,820	28,637	32,377	23,285	6574	19,722
**Scenario: Half RRSO uptake (p8 = 0.275)**														
**UK ^†^**	25.67	25.62	2546	18,589	25.66	25.61	2336	18,628	28,209	−5272	23,325	−4359	42,656	127,969
**USA ^‡^**	25.22	25.18	7271	21,982	25.22	25.17	7127	21,989	25,917	−1205	20,308	−944	57,589	172,766
**Netherlands ^∫^**	25.86	25.81	2482	24,675	25.85	25.80	2241	24,758	33,868	−11,681	27,799	−9588	50,539	151,616
**China**	20.70	20.69	820	7688	20.70	20.68	665	7568	32,321	25,018	24,651	19,081	15,531	46,592
**Brazil**	24.54	24.51	835	6319	24.53	24.49	586	6154	26,241	17,341	22,475	14,852	15,182	45,545
**India**	18.17	18.16	630	30,967	18.17	18.15	369	30,779	40,490	29,175	33,037	23,805	6574	19,722
**Scenario: Genetic testing cost of $100**														
**UK ^†^**	25.67	25.62	2443	18,468	25.66	25.61	2335	18,622	13,337	−18,988	11,070	−15,761	42,656	127,969
**USA ^‡^**	25.23	25.18	7150	21,851	25.22	25.17	7121	21,981	4717	−21,377	3718	−16,852	57,589	172,766
**Netherlands ^∫^**	25.86	25.81	2378	24,542	25.85	25.80	2238	24,749	17,893	−26,562	14,750	−21,897	50,539	151,616
**China**	20.70	20.69	721	7587	20.70	20.68	664	7567	11,165	4061	8517	3098	15,531	46,592
**Brazil**	24.54	24.51	735	6214	24.53	24.49	585	6152	14,741	6089	12,635	5219	15,182	45,545
**India**	18.17	18.16	535	30,869	18.17	18.15	368	30,778	24,832	13,577	20,267	11,081	6574	19,722

LY—life years, QALY—quality-adjusted life year, FH—family history, GDP—gross domestic product, HRT—hormone replacement therapy, ICER—incremental cost-effectiveness ratio, RRM—risk-reducing mastectomy, RRSO—risk-reducing salpingo-oophorectomy, WTP—willingness to pay. * Half the RRM uptake rate of the baseline case analysis. Baseline uptake = 47%, Half the baseline = 23.5%. ^#^ Half the RRSO uptake rate of the baseline case analysis. Baseline uptake = 55%, Half the baseline = 27.5%. **^†^** UK health-economic guideline based threshold is $28,471–$42,857/QALY. £s have been converted to $ using PPP (purchasing power parity) [28]. ^‡^ USA health-economic guideline based WTP threshold is $50,000–$100,000/QALY. ^∫^ Netherlands health-economic guideline based WTP threshold is $24,390–$60,976/QALY. €s have been converted to $ using PPP (purchasing power parity).

**Table 4:** Probability Values.

Probability	Description	Value	(95% CI) (Range)	Source
P1	BRCA1/2 mutation prevalence in general population	0.0067	(0.0059, 0.0077)	[59]
P2	Probability that carriers will undergo RRM	0.47	(0.34, 0.56)	[60]
P3	Reduction in ovarian cancer risk from RRSO	0.96	[0.8, 0.96]	[4,6]
P4	Probability of having a positive FH	0.0098	(0.0047, 0.0179)	ABCFS
P5	BRCA1/2 mutation prevalence in FH-positive individuals	0.1		[10]
P6	BRCA1/2 mutation prevalence in FH-negative individuals	0.0058	(0.0051, 0.0068)	[59], ABCFS
P7	Reduction in breast cancer risk from RRM without RRSO in BRCA1/2 carriers	0.91	(0.62, 0.98)	[8]
P8	Probability that carriers will undergo RRSO	0.55	(0.45, 0.64)	[61]
P9	Hazard ratio in breast cancer risk from RRSO alone	0.49	(0.37,0.65)	[4]
P10	Reduction in risk of breast cancer from RRM with RRSO	0.95	(0.78, 0.99)	[8]
P11	Excess CHD risk	0.0072	(0.0068, 0.0076)	[33]
P12	Fatal CHD risk	0.0303	(0.011, 0.043)	[33]
P13	Compliance with HRT	0.8	(0.76, 0.83)	[62]
P14	HR of breast cancer risk from breast cancer chemoprevention	0.71	(0.6, 0.83)	[63]
P15	Uptake of breast cancer chemoprevention	0.163	(0.136, 0.19)	[64]

95%CI—95% confidence interval, ABCFS—Australia Breast Cancer Family Study, CHD—coronary heart disease, FH—family history, RRM—risk-reducing mastectomy, RRSO—risk-reducing salpingo-oophorectomy. A detailed explanation of probabilities is given in Appendix A.

**Figure A1:**
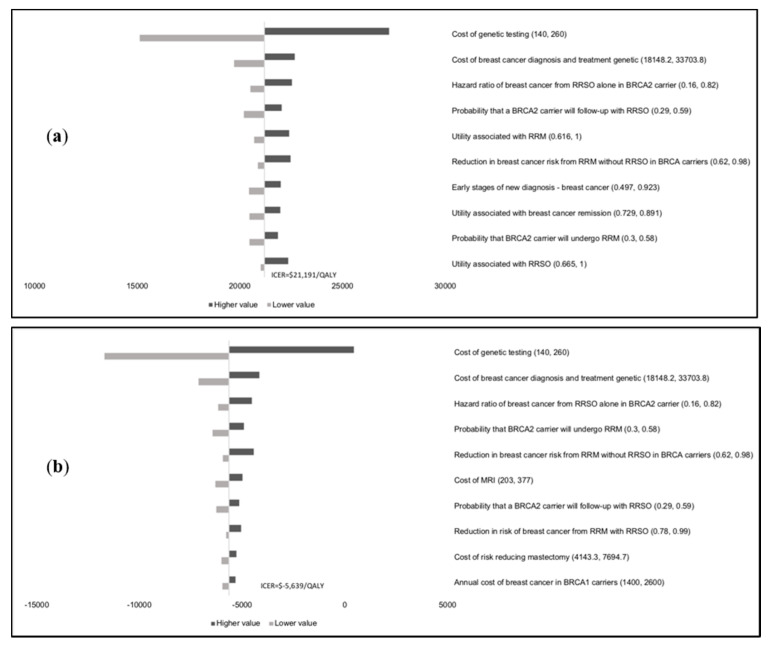
Tornado Diagram in the UK (**a**) from the healthcare payer perspective. (**b**) from the societal perspective.

**Figure A2:**
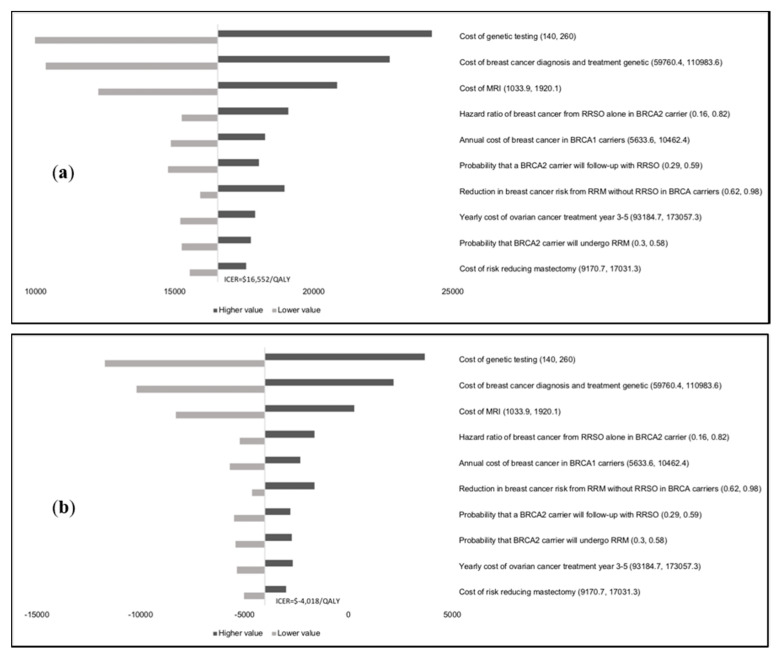
Tornado Diagram in the USA (**a**) from the healthcare payer perspective. (**b**) from the societal perspective.

**Figure A3:**
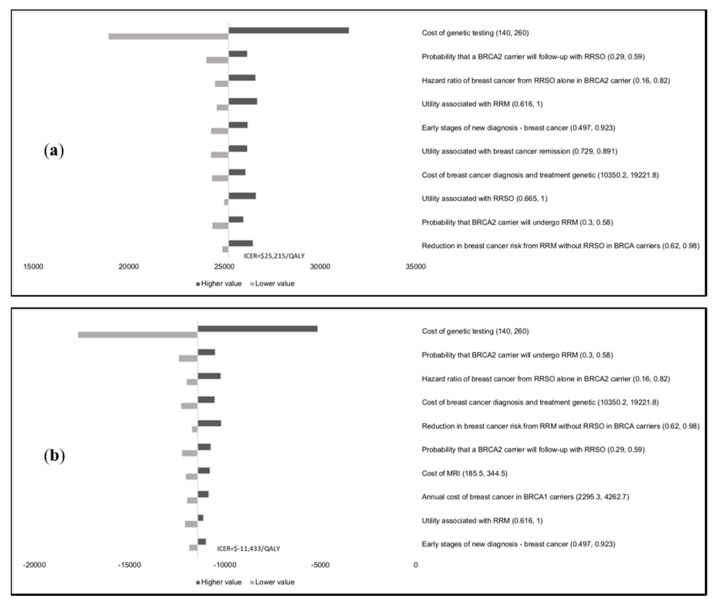
Tornado Diagram in the Netherlands (**a**) from the healthcare payer perspective. (**b**) from the societal perspective.

**Figure A4:**
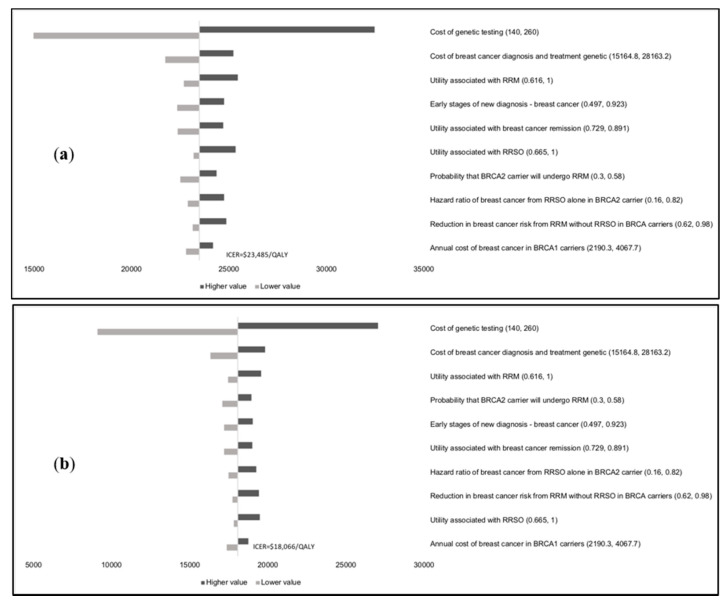
Tornado Diagram in China (**a**) from the healthcare payer perspective. (**b**) from the societal perspective.

**Figure A5:**
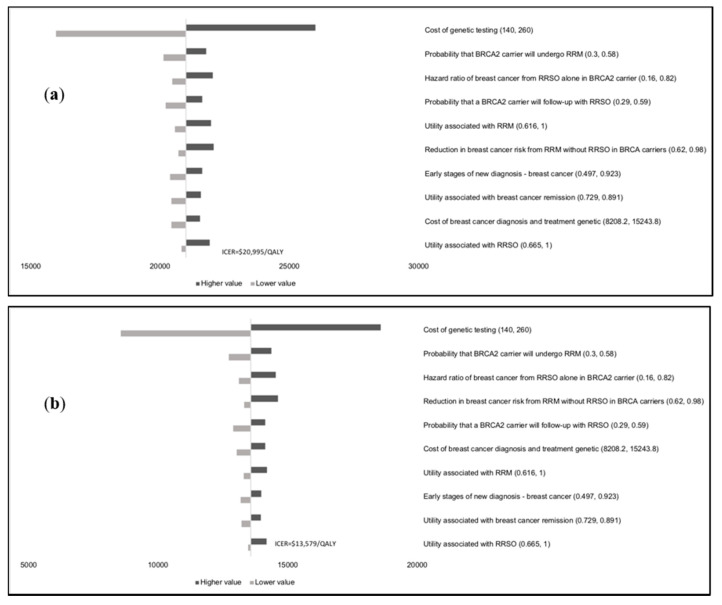
Tornado Diagram in Brazil (**a**) from the healthcare payer perspective. (**b**) from the societal perspective.

**Figure A6:**
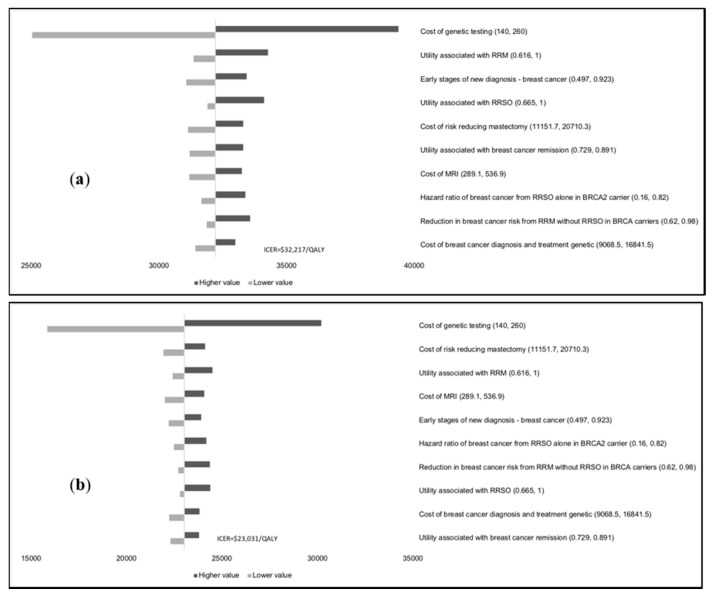
Tornado Diagram in India (**a**) from the healthcare payer perspective. (**b**) from the societal perspective.

## Appendix A. Probability Values and Explanation

**Table A1:** Probability Values.

Probability	Description	Value	(95% CI) (Range)	Source
P1	BRCA1/2 mutation prevalence in general population	0.0067	(0.0059, 0.0077)	[59]
P2	Probability that carriers will undergo RRM	0.47	(0.34, 0.56)	[60]
P3	Reduction in ovarian cancer risk from RRSO	0.96	(0.8, 0.96)	[4,6]
P4	Probability of having a positive FH	0.0098	(0.0047, 0.0179)	ABCFS
P5	BRCA1/2 mutation prevalence in FH positive individuals	0.1		[84]
P6	BRCA1/2 mutation prevalence in FH negative individuals	0.0058	(0.0051, 0.0068)	[59], ABCFS
P7	Reduction in breast cancer risk from RRM without RRSO in BRCA1/2 carriers	0.91	(0.62, 0.98)	[8]
P8	Probability that carriers will undergo RRSO	0.55	(0.45, 0.64)	[61]
P9	Hazard ratio in breast cancer risk from RRSO alone	0.49	(0.37,0.65)	[4]
P10	Reduction in risk of breast cancer from RRM with RRSO	0.95	(0.78, 0.99)	[8]
P11	Excess CHD risk	0.0072	(0.0068, 0.0076)	[33]
P12	Fatal CHD risk	0.0303	(0.011, 0.043)	[33]
P13	Compliance with HRT	0.8	(0.76, 0.83)	[62]
P14	HR of breast cancer risk from breast cancer chemoprevention	0.71	(0.6, 0.83)	[63]
P15	Uptake of breast cancer chemoprevention	0.163	(0.136, 0.19)	[64]

95%CI—95% confidence interval, ABCFS—Australia Breast Cancer Family Study, CHD—coronary heart disease, FH—family history, RRM—risk-reducing mastectomy, RRSO—risk-reducing salpingo-oophorectomy.

### Explanations

P1: *BRCA1/2* mutation prevalence in the general population is calculated based on Jervis 2015 [59].

P2: The probability that unaffected carriers will undergo RRM is taken from an analysis of UK *BRCA1/2* carriers by Evans et al. 2009 [60]. A composite uptake rate for *BRCA1* (60% RRM rate) and *BRCA2* (43% RRM rate) carriers weighted for the relative prevalence of *BRCA1* and *BRCA2* mutations was computed [60].

P3: The reduction in ovarian cancer risk obtained from RRSO is taken from previous studies which report a 4% residual risk of primary peritoneal cancer following RRSO [6].

P4: The probability of having a positive family history in general population is obtained from the Australia Breast Cancer Family Study (ABCFS).

P5: The overall *BRCA1/BRCA2* mutation prevalence (10%) among FH-positive breast cancer patients is based on the current testing guideline.

P6: The *BRCA1/2* mutation prevalence in FH negative individuals is calculated based on the *BRCA1/2* mutation prevalence in the general population, the BRCA1/2 mutation prevalence in FH-positive individuals, and the probability of having a positive FH.

P7: The reduction in breast cancer risk from RRM in *BRCA1/BRCA2* mutation carriers not undergoing RRSO is taken from the PROSE study data by Rebbeck et al. 2004 [8].

P8: The uptake of RRSO in unaffected *BRCA1/BRCA2* carriers is taken from a study among high-risk UK women [7].

P9: The hazard ratio for breast cancer in premenopausal unaffected *BRCA1/BRCA2* women undergoing RRSO alone is taken from a meta-analysis by Rebbeck et al. 2009 [4].

P10: The reduction in breast cancer risk in *BRCA1/BRCA2* mutation carriers undergoing RRM and RRSO is taken from the PROSE study data by Rebbeck et al. 2004 [8].

P11: Excess risk of CHD after RRSO is estimated using data from Parker 2013 [33]. The absolute excess CHD incidence is obtained by subtracting CHD incidence in women undergoing RRSO from those who have not.

P12: The risk of CHD mortality is obtained from the Nurses Health Study (Parker et al. 2013) [33]. Death from CHD is reported in 1 in 33 premenopausal women undergoing RRSO and not taking HRT [33].

P13: HRT compliance rate is obtained from a UK cohort (Read et al., 2010) [62].

P14: The Hazard Ratio for breast cancer risk from chemoprevention in high-risk women is obtained from the extended long-term follow-up of the IBIS-I breast cancer prevention trial (Cuzick et al. 2015) [63].

P15: The uptake of breast cancer chemoprevention is obtained from a recent meta-analysis by Smith et al. 2016 [64].

## Appendix B. Medical Costs in 2016 Values (USA Dollars Converted by PPP)

**Table A2:** Medical costs in 2016 values (USA dollars converted by PPP).

Cost descriptions	UK		US	Netherlands		China		Brazil		India	
	GBP	USD	USD	EUR	USD	RMB	USD	BRL	USD	INR	USD
Cost of genetic testing		200	200		200		200		200		200
Cost of genetic counselling	29	42	42	55	67	0	0	135	68	733	42
Cost of prophylactic bilateral salpingo-oophorectomy	2799	3999	7904	3713	4584	4525	1308	957	483	82,368	4712
Cost of ovarian cancer diagnosis and treatment	14,268	20,383	133,121	23,238	28,689	12,991	3755	12,564	6345	613,662	35,107
Annual cost of ovarian cancer in years 1 to 2	5433	7761	14,635	10,865	13,413	48,495	14,016	4442	2244	290,086	16,595
Annual cost of ovarian cancer in years 3 to 5	5090	7271	14,635	10,480	12,939	48,021	13,879	4278	2161	280,720	16,059
Terminal care cost with ovarian cancer	16,452	23,503	93,005	11,325	13,981	10,060	2907	1358	686	80,623	4612
Cost of risk reducing mastectomy	4143	5919	13,101	2950	3642	2634	761	867	438	278,474	15,931
Annual cost of hormone replacement therapy	60	86	52	61	76	2148	621	217	110	15,595	892
Cost of mammography	60	85	156	95	117	82	24	42	21	2051	117
Cost of MRI	203	290	1477	215	265	605	175	252	127	7222	413
Cost of breast cancer diagnosis and treatment in general population	18,148	25,926	85,372	11,977	14,786	74,959	21,664	23,218	11,726	226,451	12,955
Annual cost of breast cancer in general population	1388	1982	8048	2718	3355	12,360	3572	2328	1176	55,519	3176
Cost of breast cancer diagnosis and treatment in BRCA1/2 carriers	16,499	23,570	78,964	10,780	13,309	68,476	19,791	20,861	10,536	200,902	11,493
Annual cost of breast cancer in BRCA1/2 carriers	1400	2000	8048	2656	3279	10,827	3129	1999	1009	53,959	3087
Terminal care cost with breast cancer	16,452	23,503	68,022	11,325	13,981	10,060	2907	1358	686	80,623	4612
Cost of fatal coronary heart disease	3387	4839	23,934	3008	3714	11,972	3460	2953	1491	47,673	2727
Annual cost of excess coronary heart disease	122	175	7277	109	134	526	152	124	63	3708	212
Annual cost of chemoprevention	19	27	899	36	45	93	27	499	252	62	4

CHD—coronary heart disease, HRT—hormone replacement therapy, MRI—magnetic resonance imaging, RRM—risk-reducing mastectomy, RRSO—risk-reducing salpingo-oophorectomy, PPP—purchasing power parity.

### Appendix B.1. Explanations

All costs are adjusted for 2016 consumer price index.

For comparison, we convert values in all other country currencies (£s, €s, ¥, ₹, R$) to $ (USA) using purchasing power parity (PPP) factor [28].

We collected primary data on relevant direct medical costs from the Urban Basic Medical Insurance Database in China [65]; the Dutch Healthcare Authority (NZA) in Netherlands; Management System of Procedures/Medical drugs/Orthotics/Prosthetics/Special Materials (SIGTAP) [66], the Health Price Bank (BPS) [67] and Chamber of Regulation of the Market of Medicines (CMED) [68] in Brazil; and an accredited Cancer Centre (Tata Medical Centre) in India. UK costing data were obtained from published NHS reference costs for the UK [69,70].

### Appendix B.2. Cost of Genetic Testing/Counselling

We use a standard international cost for genetic testing for all countries (US$ 200 in 2016). We assume a 71% uptake of genetic testing (based on our previous population based research studies) [22]. All participants have pre-test counselling and post-test counselling is received by those testing positive (pathogenic/likely pathogenic carriers). We assume a VUS prevalence of 2% and include the cost of post-test counselling for VUS in these 2% cases [54].

The cost of *BRCA1/BRCA2* testing is based on testing costs for these genes in our population testing research programme as well as confirmatory testing costs in an accredited national genetics laboratory for those testing positive. The UK national unit cost assumed for genetic counselling is £44 per hour of client contact from PSSRU Unit costs of Health and Social Care 2010 [22,85,86]. The US genetic counselling costs are obtained from Schwartz 2014 and include ancillary preparation (scheduling/administration), counsellor preparation, and counselling [87]. The genetic counselling costs in the Netherlands, Brazil, and India were obtained from primary data. There is no additional physician genetic counselling cost charged from patients in China; hence, this was not incorporated for Chinese analysis.

### Appendix B.3. RRSO Costs

The UK RRSO costs are obtained from NHS reference costs [88], and the US costs are from Grann 2011 [89] inflated using the medical component of the USA consumer price index to 2016 US$. Costs of HRT for the UK are taken from BNF [90] and for the USA from William-Frame 2009 [91]. The costs of RRSO and HRT in Netherlands, China, Brazil, and India are obtained from primary data. Costs assume HRT is given from average age of RRSO to the average age of menopause (51 years). These costs are calculated for the 80% assumed to be compliant with HRT. Costs include the cost of three follow-up DEXA scans for monitoring bone health and calcium and vitamin-D3 for additional osteo-protection.

### Appendix B.4. RRM

The UK RRM costs are obtained from NHS reference costs [88], and the USA costs are from Grann 2011 [89] inflated using the medical component of the US consumer price index to 2016 US$. The RRM costs in Netherlands, China, Brazil and India are obtained from primary data.

### Appendix B.5. Costs of Ovarian Cancer

We assume that the costs of ovarian cancer diagnosis include a pelvic examination, ultrasound scan, CA125 test, CT scan, percutaneous biopsy, and peritoneal cytology. The costs of ovarian cancer treatment include the reference cost for a lower and upper genital tract very complex major procedure and administration of chemotherapy based on 6 cycles of carboplatin and paclitaxel treatment. It is assumed that in the first and second years, treated survivors would have a further three consultant visits, a CT scan, and four CA125 tests each year. In the third to fifth years post-surgery, it is assumed that survivors would have two consultant visits and two CA125 tests.

Costs for ovarian cancer diagnosis and treatment in the UK are derived from national reference costs and a recent ovarian cancer guideline developed by NICE [88,92]. Annual costs of ovarian cancer treatment in the USA are taken from Grann et al. 2011 [89] and inflated using the medical component of the USA consumer price index to 2016 US$. We include the costs of treatment of recurrence taken from Cancer Research UK [93] and Grann 2011 [89]. The costs of ovarian cancer diagnosis and treatment in Netherlands, China, Brazil, and India are obtained from primary data.

The costs of ovarian cancer terminal care are derived from end-of-life costs for cancer patients based on a report from the National Audit office UK [94]. For the USA, the terminal care costs for ovarian cancer are obtained from Grann 2011 [89], which were inflated using the medical component of the USA consumer price index to 2016 US$. The costs of ovarian cancer terminal care are obtained from primary data in the Netherlands, China, Brazil and India. In line with NICE recommendations, future healthcare costs not associated with ovarian cancer are not considered [95].

### Appendix B.6. Costs of Breast Cancer

In the general population, 10% breast cancer is non-invasive DCIS and 90% is invasive. 95% of invasive breast cancer is early and locally advanced (stages 1–3), and 5% of invasive breast cancer is advanced breast cancer (stage 4) [96]. In *BRCA1/2* carriers, 20% of cancers are DCIS and 80% are invasive [9,97].

Seventy percent of invasive breast cancers are ER-positive [98,99], among which 49% are premenopausal; 15% of early/locally advanced breast cancers and 25% of advanced breast cancers are HER2-positive; 27% *BRCA1* and 67% *BRCA2* breast cancers are ER-positive; 5% *BRCA1* and 14% *BRCA2* breast cancers are HER2-positive [100,101,102,103,104,105]. All costs are adjusted for *BRCA1/BRCA2* breast cancers for differences in stage at presentation, the proportion of being non-invasive, and the proportion of being ER-positive or HER2-positive.

Annual breast cancer treatment costs in the USA are obtained from Grann et al. 2011 [89] and inflated using the medical component of the USA consumer price index to 2016 US$. In the UK, Netherlands, China, Brazil, and India, breast cancer treatment costs are estimated based on clinical guidelines and unit costs are detailed as below.

Diagnosis costs: Whether suspected at breast screening or through presentation to the GP, diagnosis in the breast clinic is made by triple assessment (clinical assessment, mammography, and ultrasound imaging with core biopsy and/or fine needle aspiration cytology) [98]. Clinical examination and mammography costs are from the paper by Robertson C et al. [106]. Breast ultrasound and biopsy costs are obtained from NHS reference costs [88] in the UK and from primary data in Netherlands, China, Brazil, and India. For all patients presented with suspected advanced breast cancer, MRI should be offered to assess for bone metastases [99].

Sentinel lymph node biopsy (SLNB) costs: SLNB is used for staging axilla for early invasive breast cancer and no evidence of lymph node involvement on ultrasound or a negative ultrasound-guided needle biopsy (73% of early and locally advanced invasive cancers). The SLNB costs in the UK are obtained from NHS reference costs including sentinel lymph node scan and unilateral intermediate breast procedures [88]. The SLNB costs in Netherlands, China, Brazil, and India are obtained from the primary data sources described above.

Pretreatment axilla ultrasound costs: Pretreatment ultrasound evaluation of the axilla should be performed for all patients being investigated for early invasive breast cancer and, if morphologically abnormal lymph nodes are identified, ultrasound-guided needle sampling should be offered [96]. The commissioning cost of pretreatment ultrasound evaluation of the breast and axilla is the same as that of the breast only [88]. The costing model considers the cost of ultrasound-guided needle sampling only, obtained from NHS reference costs (UK) [24] and primary data (Netherlands, China, Brazil, and India).

Axillary lymph node dissection (ALND) costs: ALNB is undertaken for lymph node positive cancers (approximately 31% early and locally advanced invasive cancers—NICE guideline and BCCOM project [96,98,107]; 30% node positive for BRCA1/2 breast cancer—familial breast cancer screening studies, breast cancer case series and Early Breast Cancer Trialists’ Collaborative Group data) [97,100,101,102,108]. The cost of ALND is assumed to be 25% of the cost of breast surgery as per NICE guideline development group recommendations [96].

Breast surgery costs include costs of breast-conserving surgery (assumed for all non-invasive cancers and 75% of early/locally advanced invasive cancers) and costs of mastectomy with reconstruction (for 25% early/locally advanced and all advanced cancers). Costs are obtained from the national NHS reference costs (UK) [88] and primary data (Netherlands, China, Brazil, and India).

Chemotherapy and radiotherapy costs: Invasive breast cancers who are not at low risk [107,109,110] receive adjuvant treatment in line with NICE guidelines. Costs include radiotherapy costs for 60% of early invasive/locally advanced, radiotherapy, and chemotherapy costs for 40% early invasive/locally advanced, and chemotherapy for all advanced cancers. Radiotherapy costs include planning and 40Gy in 15 fractions over 3 weeks [98] or palliative treatment; these were taken from national NHS reference costs [88]. Chemotherapy costs based on polychemotherapy [108] include administration costs, the costs of first and second-line therapy and toxicity from NICE guidelines [96,99]. In the Netherlands, China, Brazil, and India, radiotherapy costs and chemotherapy costs are obtained from the primary data sources described above.

Endocrine therapy costs: As per NICE guidelines [96,98], ER-positive invasive breast cancers receive Tamoxifen 20 mg/day (premenopausal) or Anastrazole 1mg/day (postmenopausal). Seventy percent of invasive breast cancers are ER-positive [98,99], among which 49% are premenopausal. We assume that the length of endocrine therapy is 5 years. The drug costs are obtained from the BNF [26] in the UK. ER testing costs are obtained from a local NHS trust and included for all invasive breast cancers. The costs of drugs and ER testing are obtained from primary data sources in the Netherlands, China, Brazil, and India described above.

Target therapy costs: HER2-positive breast cancer patients can be given at 3-week intervals for 1 year or until disease recurrence as per NICE guidelines. Breast cancer patients with positive HER2 are eligible for treatment with trastuzumab [98,99]. Ten percent of the eligible patients are intolerant of trastuzumab. Among women suitable for this treatment, 80% receive trastuzumab [96]. HER2 testing costs are obtained from a local NHS trust and included for all invasive breast cancers. The trastuzumab cost per patient including the administration of treatment and cardiac monitoring is £15080, which was obtained from the NICE costing report [96]. In the Netherlands, China, Brazil, and India, the costs of HER2 testing and trastuzumab are obtained from the primary data sources described above.

Follow-up costs: Breast cancer patients are offered mammographic surveillance and clinical follow up, with the screening cost of £141.45 per women in 2011 [106]. We assume that patients are followed up every four months in the first two years, every six months from the third to the fifth year, and every year from the sixth to the 10th year.

Bisphosphonate costs: Bisphosphonates is considered to be offered to patients newly diagnosed with bone metastases to prevent skeletal-related events and reduce pain [99]. Seventy-four percent of patients with advanced breast cancer will develop bone metastases, and 65% of patients with bone metastases are offered bisphosphonates [96,111]. Bisphosphonates that are currently offered include oral sodium clodronate, ibandronic acid, zoledronic acid, and pamidronate. The proportions of patients receiving the four drugs are 20%, 30%, 25%, and 25%, respectively. The annual costs including administration for the four drugs are £1971, £2541.96, £3208, and £3208 respectively, which were obtained from the NICE costing report [96]. We assume that the average length of bisphosphonates treatment is 2.7 years, which is the life expectancy of advanced breast cancers based on one-year survival rate (63.2%) [112]. The bisphosphonate costs in the Netherlands, China, Brazil, and India are obtained from the primary data sources described above.

Recurrence costs: For non-invasive breast cancers, the non-invasive and invasive relapse rates are both 12.5%. Thirty-five percent of early and locally advanced invasive breast cancers progress to advanced disease [96]. The recurrence rates for early and locally advanced breast cancer are 15.9% for node-positive [113] and 11% for node-negative disease [114]. Weighted for 31% node positive and 69% node negative, the composite recurrence rate for early and locally advanced breast cancer is 12.5%. The recurrence rate for the advanced disease is 66% (34% relapse-free five-year survival) [115].

Terminal care costs: The costs of terminal care for breast cancer are derived from end-of-life costs for cancer patients based on a report from the National Audit office UK [30]. For the US, the terminal care costs for breast cancer are obtained from Grann 2011 [89], and these were inflated using the medical component of the US consumer price index to 2016 US$. The costs of breast cancer terminal care are obtained from primary data sources in the Netherlands, China, Brazil, and India. In line with NICE recommendations, future healthcare costs not associated with breast cancer were not considered [95].

### Appendix B.7. Cost of Breast Cancer Screening

For non-carriers, we assume routine triennial mammography between 50 and 70 years as per the UK NHS breast cancer screening programme [116] (seven mammograms on average). Breast screening in the USA assumes mammography every two years starting at 50 years [117]. In the Netherlands, the National Breast Cancer Screening Programme is designed for women between 50 and 75 years of age. Once every 2 years, women in this age group are invited for a mammogram. The guidelines from the Brazilian Ministry of Health is for all women aged 50–69 years to be screened with mammography only every 2 years. The coverage in the target age group remains low ranging from 27% to 51% [118]. To obtain a conservative estimate of the cost-effectiveness of population-based genetic testing, we adopted the highest value of uptake (51%) in Brazil. There is no national breast cancer screening programme in China or India.

For BRCA1/BRCA2 mutation carriers, we assume an annual mammogram from 40 to 69 years and annual MRI from 30–49 years as per NICE guidelines for familial breast cancer [119] (30 mammograms and 20 MRIs on average). We assume that breast cancer screening policies for BRCA1/2 carriers in the Netherlands, China, Brazil, and India, are the same as that in the UK. For the USA, it is based on annual mammography and MRI starting at 30 years, and annual mammography only from age 50 years [117].

### Appendix B.8. Cost of Chemoprevention

*BRCA1/BRCA2* mutation carriers are offered tamoxifen (premenopausal) or raloxifene (postmenopausal) for 5 years [119,120] to reduce breast cancer risk. The drug costs are obtained from the BNF (UK) [90], Grann 2011 (US) [89], and primary data (Netherlands, China, Brazil, and India). A 16.3% uptake is assumed for chemoprevention [64].

### Appendix B.9. Cost of CHD

Cost of excess CHD: British Heart Foundation statistics reports costs per capita across four commissioning regions in England (London, Midlands and East, North, and South) [121].

The costs of CHD and stroke are averaged across the four regions. The prevalence of CHD is estimated at 12.0% in the UK [121] and 11.7% in the USA [122], with the onset of CHD estimated at 55 years of age [33,123].

The yearly cost of CHD in the UK is obtained by dividing the per capita cost by the population prevalence of CHD [121]. Using the report published by the American Heart Association [124], the total cost of CHD, CHF, and stroke were divided by the population with CHD [122,125], giving the yearly cost of CHD in the USA. This yearly cost is multiplied by the number of years between onset of CHD and average life expectancy to provide the cost attributed to excess CHD.

Cost of fatal CHD: This is costed on the basis of a fatal myocardial infarction using NHS reference costs [88]. USA costs are obtained from Afana et al. 2015 [126], and these are inflated using the medical component of the US consumer price index to 2016 US$.

We used the ratio of breast cancer treatment costs in the Netherlands, China, Brazil, and India compared to treatment costs in the UK to impute the costs of excess CHD and fatal CHD in each of these countries (Netherlands, China, Brazil, and India) based on the cost of CHD in the UK.

## Appendix C. Estimation of Productivity Loss

The retirement ages for females are 65 in the UK, 62 in the USA, 50–55 in China, 60 in Brazil, 68 in Netherlands, and 60–65 in India. We used the lower values of the retirement age ranges in China and India to get the conservative estimates of productivity loss. The female labour force participation rates are 56.77% in the UK, 55.99% in the USA, 62.03% in China, 53.32% in Brazil, 58.02% in Netherlands, and 27.45% in India, which were obtained from the World Bank [71]. The hourly wage rage across countries are presented in Table A3.

**Table A3:** Hourly wage rage across countries (USA dollars in 2016).

Age	UK	USA	Netherlands	China	Brazil	India
30–34	19.47	13.08	16.85	5	5.54	4.77
35–39	19.47	14.75	22.37	5	5.54	4.58
40–44	19.33	14.75	22.37	5	5.54	4.58
45–49	19.33	14.97	24.11	5	5.54	6.56
50–54	17.42	14.97	24.11		5.54	6.56
55–59	17.42	15.10	24.19		5.54	3.71
60–64	15.08	15.10	24.19			
65–69			21.32			
Source	[127]	[128]	[129]	[130]	[131]	[132]

We categorised the productivity costs as three subcomponents: (1) temporary disability due to short-term work absences following diagnosis, (2) permanent disability due to reduced working hours following a return to work or workforce departure; and (3) premature mortality due to death before retirement [72], as detailed in Table A4.

**Table A4:** Descriptive statistics for productivity loss in breast and ovarian cancer patients.

Variables	Breast Cancer	Ovarian Cancer
(1) Temporary disability		
Percentage of temporary disability cases	94.0%	98% ^1^
Average time taken off work following diagnosis (weeks)	44.9	47.22 ^2^
(2) Permanent disability		
Percentage of permanent disability: reduced hours	26%	40% ^3^
Reduced hours per week after returning to work (hours)	5.5	5.5
(3) Premature mortality (before retirement)		
Percentage of permanent disability: workforce departure	12.9%	30% ^3^

Source: Hanly P, et al., 2012 [72]. ^1^ We assume 98% ovarian cancer patients have cancer-related short-term work absences after diagnosis. ^2^ We assume ovarian cancer patients experience four weeks for surgery, 24 weeks for chemotherapy, and 24 weeks for recurrence treatment with the recurrence rate of 80% [133]. ^3^ We assume the percentages of permanent disability for ovarian cancer are 40% for reduced working hours and 30% for workforce departure.

We estimated temporary disability as time absent from work multiplied by age-specific gross earnings.

We calculated productivity costs due to permanent disability by applying age-specific gross earnings to the reduction in working hours, or the number of working hours if permanent workforce departure, until retirement age. Regarding productivity loss from premature mortality, we assumed that without cancer, the productive capacity of an individual would continue from the age of diagnosis until age of retirement. We multiplied the projected years of life lost by the age-specific gross earnings for the remainder of the working life to generate monetary estimates.

## Appendix D. Maximum Values of Genetic Testing Costs at Which Offering Genetic Testing for the Population Remains Cost-Effective.

**Table A5:** Maximum values of genetic testing costs at which offering genetic testing for the population remains cost-effective.

	Payer Perspective		Societal Perspective	
	Lower WTP ^#^	Higher WTP^##^	Lower WTP ^#^	Higher WTP ^##^
**Thresholds based on GDP**				
UK	$412 ($42,648/QALY)	$1254 ($127,869/QALY)	$677 ($42,639/QALY)	$1520 ($127,960/QALY)
USA	$519 ($57,490/QALY)	$1417 ($172,735/QALY)	$680 ($57,582/QALY)	$1577 ($172,698/QALY)
Netherlands	$442 ($50,539/QALY)	$1407 ($151,520/QALY)	$792 ($50,517/QALY)	$1758 ($151,603/QALY)
China	$146 ($15,402/QALY)	$354 ($46,536/QALY)	$183 ($15,522/QALY)	$390 ($46,506/QALY)
Brazil	$130 ($15,143/QALY)	$493 ($45,490/QALY)	$219 ($15,168/QALY)	$582 ($45,515/QALY)
India	Not cost-effective	$95 ($19,670/QALY)	$62 ($6,540/QALY)	$172 ($19,685/QALY)
**Thresholds based on local economic evaluation guidelines**				
UK	$238 ($28,386/QALY)	$365 ($42,826/QALY)	$481 ($28,406/QALY)	$608 ($42,845/QALY)
USA	$460 ($49,919/QALY)	$850 ($99,969/QALY)	$620 ($49,882/QALY)	$1010 ($99,933/QALY)
Netherlands^∫^	$293 ($24,364/QALY)	$800 ($60,934/QALY)	$582 ($24,369/QALY)	$1089 ($60,939/QALY)

^#^ 1*GDP per capita, ^##^ 3*GDP per capita, WTP—willingness to pay (threshold), GDP—gross domestic product.

The appendix describes the maximum genetic testing costs and corresponding ICER/QALY (in brackets) at which offering *BRCA* testing for the population will remain cost-effective. Results are presented for both the payer and societal perspectives.

For GDP-based thresholds: This is cost-effective at the standard 3*GDP per capita WTP threshold and highly cost-effective at the 1*GDP per capita WTP threshold [82]. The discount rate is 3% for costs and health effects (LYs and QALYs) [81].

For country-specific thresholds:

For the UK, this is £20,000 to £30,000 [25,134]; for the USA, this is $50,000 to $100,000 [26,135]; for the Netherlands, this is: €20,000 to €50,000 [27]. Values in £s and €s have been converted to $ using PPP (purchasing power parity) [28].

For country-specific thresholds:

For the UK, the discount rate is 3.5% discount for costs and QALYs [25,134]; for the USA, this is 3% discount for costs and QALYs [53]; for the Netherlands, this is 4% discount for costs and 1.5% discount for QALYs [136].

Perspective:

WHO guidelines recommend a societal perspective [81,82].

Dutch guidelines recommend a societal perspective [136]. UK NICE guidelines recommend a payer perspective [25]. US guidelines recommend presentation of both societal and payer perspectives [53].

## Appendix E. One-Way Sensitivity Analyses

One-way sensitivity analysis for all probabilities, costs, and utilities in terms of ICER of population-based BRCA testing compared to a clinical-criteria/FH-based approach in the UK, USA, Netherlands, China, Brazil, and India from both the payer perspective and the societal perspective.

X-axis: Incremental cost-effectiveness ratio (ICER): cost (£s or $s) per quality-adjusted life year (QALY) (discounted).

Y-axis: Probability, cost, and utility parameters in the model. The model is run at both lower and upper values/limits of the 95% confidence interval or range of all probability parameters described in Table 1, and both lower and upper values/limits of the cost and utility-score parameters given in the methods and Table 2.

‘Upper value’ represents outcomes for the upper limit of the parameter, and ‘Lower value’ represents outcomes for lower limit of the parameter.
